# Harrison’s rule corroborated for the body size of cleptoparasitic cuckoo bees (Hymenoptera: Apidae: Nomadinae) and their hosts

**DOI:** 10.1038/s41598-022-14938-9

**Published:** 2022-06-29

**Authors:** Kayun Lim, Seunghyun Lee, Michael Orr, Seunghwan Lee

**Affiliations:** 1 Insect Biosystematics Laboratory, Department of Agricultural Biotechnology, Seoul National University, Seoul, 08826 Republic of Korea; 2 Key Laboratory of Zoological Systematics and Evolution, Institute of Zoology, Chinese Academy of Sciences, 92 Box, No. 1 Beichen West Road, Chaoyang District, Beijing, 100101 People’s Republic of China; 3 Research Institute of Agriculture and Life Sciences, Seoul National University, Seoul, 08826 Republic of Korea

**Keywords:** Evolution, Zoology

## Abstract

Harrison’s rule, that body size is positively correlated between parasites and hosts, has been reported in a range of taxa, but whether the rule is applicable to cleptoparasitic insects is poorly understood. Subfamily Nomadinae, the largest group of cleptoparasitic bees, usurp the nests of a variety of host bees. Within the subfamily, *Nomada* exploits the most diverse hosts, using at least ten genera from five families. Here, we reassess the phylogeny of Nomadinae, including the expanded sampling of the genus *Nomada*, to explore host shift fluctuations throughout their evolutionary history and test the applicability of Harrison’s rule for the subfamily. Our phylogenetic results are mostly congruent with previous investigations, but we infer the tribe Hexepeolini as a sister taxon to the tribe Nomadini. Additionally, the results reveal discrepancies with the traditional classifications of *Nomada*. Ancestral state reconstruction of host use indicates that, early in their evolution, parasites used closer relatives, before attacking less related groups later. Lastly, we confirm Harrison’s rule in Nomadinae, supporting that body size dynamics influence the host shifts of cleptoparasitic bees.

## Introduction

Body size influences many aspects of organismal biology, such as patterns of resource use^1–3^, habitat colonization potential^4,5^, and ecological strategy^6,7^. Among species with strong ecological interactions, body size can play an even stronger role, and this is especially important for parasites and their hosts^8^. The importance of size in parasitism is highlighted in Harrison’s rule (HR), which states that host and parasite body sizes generally positively covary^9^.

Given that parasite body size depends on that of their hosts to some degree, we must explore the evolutionary trends of host-parasite association to test for morphological similarity and divergence between parasites and hosts. Although HR is a common pattern across the animal kingdom, it remains poorly understood from a macroevolutionary viewpoint; the rule has been mostly demonstrated using phylogenetically-independent comparisons to date (i.e. simple allometry between hosts and parasites^10–15^,). Exploring HR under a molecular phylogenetic framework may offer new insights into the underlying adaptive basis and historical context^16^, while also providing invaluable quantitative metrics of relatedness. For example, are body size correlations a result of co-speciation or does it largely result from shifts to a host that has a similar body size? For such a study, it is ideal to focus on a monophyletic lineage of obligatory parasites with both high body size variation and good host-parasite association data.

Also, HR has been documented in many parasitic organisms: parasitic nematodes and their hosts^12^, parasitic barnacles and decapods^17^, avian lice and birds^18^, and fleas and rodents^19^. HR has been tested for several insect groups previously, in terms of both traditional parasitism and herbivory, but most insect groups remain unexplored^11^. One parasitic organism for which HR has never been extensively and quantitatively tested is the cleptoparasitic bees. About 13% of all bees and 20% of bees in the family Apidae exhibit the cleptoparasitic lifestyle^20^. Rozen^21^ indirectly proposed that HR applies to these bees, but quantitative analyses have yet to be conducted.

Cleptoparasitic bees secretly enter the nests of other bees and lay their eggs near or in the pollen balls that the hosts provisioned for their offspring, and either the cleptoparasitic female or the cleptoparasitic brood removes the host larva or egg^22,23^. The larvae consume the pollen that the host female prepared, pupate, and emerge from the host bee nest^24^. Body size may strongly affect the interaction between cleptoparasitic bees and their hosts in that i) nest entrance size physically constrains the width of parasitic females; ii) cell size physically limits the development of offspring; iii) the amount of the food source depends on the hosts and is generally correlated to the size of the host^25^; and iv) in the event of conflict, physical size may influence who is the victor. Consequently, cleptoparasitic bees are generally expected to only parasitize similar or smaller host bees on the individual level (physical constraint: i, ii) but, at the same time, it is advantageous to evolve to a similar size of the hosts to maximally utilize the food source and defend themselves (physiological constraint: iii, conflict constraint: iv). These considerations suggest that HR should be corroborated in cleptoparasitic bees, although they may also be slightly smaller, warranting formal testing.

We selected the cuckoo bee subfamily Nomadinae sensu Bossert et al. 2019^26^ (Nomadinae hereafter) as a model group for this study. Nomadinae, with nearly 1,300 described species globally, is the largest lineage where all members are obligatory brood parasites^27^. They have repeatedly been recovered as a monophyletic group using several different datasets including Sanger multi-locus data^28–30^, transcriptomes and UCEs^26^, and UCEs alone^31^. Nomadinae represent an ancient origin of cleptoparasitism (c.a. 100 mya ^28,31^,) and they attack a wide variety of bees regardless of phylogenetic affinity (Andrenidae, Apidae, Colletidae, Halictidae, and Melittidae). A great deal of work has been done on determining hosts in the Nomadinae^32–35^. Alongside this, the nesting behaviors and immature biology of multiple genera have been meticulously investigated^21,36,37^. In Nomadinae, genera in the same tribe might infiltrate hosts in different families, while genera in different tribes might utilize exclusively the same host genera^24^. Remarkably, even small genera may use entirely different families (four species on two families, *Townsendiella*^38^).

Species that belong to the tribe Nomadini have the largest host range, using 10 genera of five families as hosts^20^; at the other end of the spectrum, the large genus *Epeolus* (tribe Epeolini) attacks only the genus *Colletes*^39^. Even though the tribe Nomadini has the highest species diversity and the vast host information, its phylogenetic relationships have not been entirely resolved. Following prior works on the group, Alexander conducted species-group classification in the genus *Nomada*, which is the sole genus in the tribe Nomadini, based on cladistic analyses^40^. There, he defined 16 species-groups, and this classification has been commonly used since^35,41–43^. However, he mentioned that one of the species-groups, the *ruficornis* species-group may be a paraphyletic due to the lack of distinct apomorphic characteristics. Recently, the incongruencies between the morphological study and molecular analysis was proposed by Won, and Odanaka et al.^44,45^. Yet, given its high species diversity in this tribe, it is crucial to reveal the hidden patterns of the phylogenetic relationships.

The majority of cuckoo bees are specialists in that they parasitize only one or a few relative hosts in the same genus^25^, although there are exceptions such as the more generalist genus *Sphecodes* in the family Halictidae. There are numerous ways in which cleptoparasitic bees have adapted to track their hosts, including phenological synchrony, and they can also exhibit similar spatial richness patterns^46^. Nomadine bees also vary greatly in size, making them ideal for studies on HR: the body length of one of the largest species (*Acanthopus excellens* Schrottky, 1902; ~ 23 mm) is more than ten times larger than one of the smallests (*Oreopasites barbarae* Rozen, 1992; 2.2 mm ~)^36,47^. Altogether, these traits provide a unique opportunity to explore HR in a molecular phylogenetic framework.

This study empirically tests HR in the cleptoparasitic bee lineage Nomadinae to infer the role of body size in their macroevolutionary dynamics for the first time. At the same time, we also briefly explore how closely related parasites and hosts are in this group, as Emery’s rule predicts that parasites should be closely related to their hosts^48^. We measured the body size of specimens, retrieved data from the literature (see Supplementary Data 1), and conducted allometric analyses of Nomadinae and their hosts. Then we revisited the phylogeny of Nomadinae with an increased sampling of the genus *Nomada*, the largest genus of cleptoparasitic bees^35^. Finally, we conducted ancestral state reconstruction of host associations and body size, using the resulting phylogenetic tree to infer their evolutionary history and correlation between the two traits (with focus on species whose hosts are known).

The main questions explored by this study are as follows: i) Does body size covary between hosts and parasites?; ii) How does body size change across the phylogeny, and do major shifts correlate with phylogeny or with host switches?; iii) How does host-specificity evolve across the tree and do nomadine bees follow Emery’s rule?

## Results

### Phylogenetic analysis

**Figure 1:**
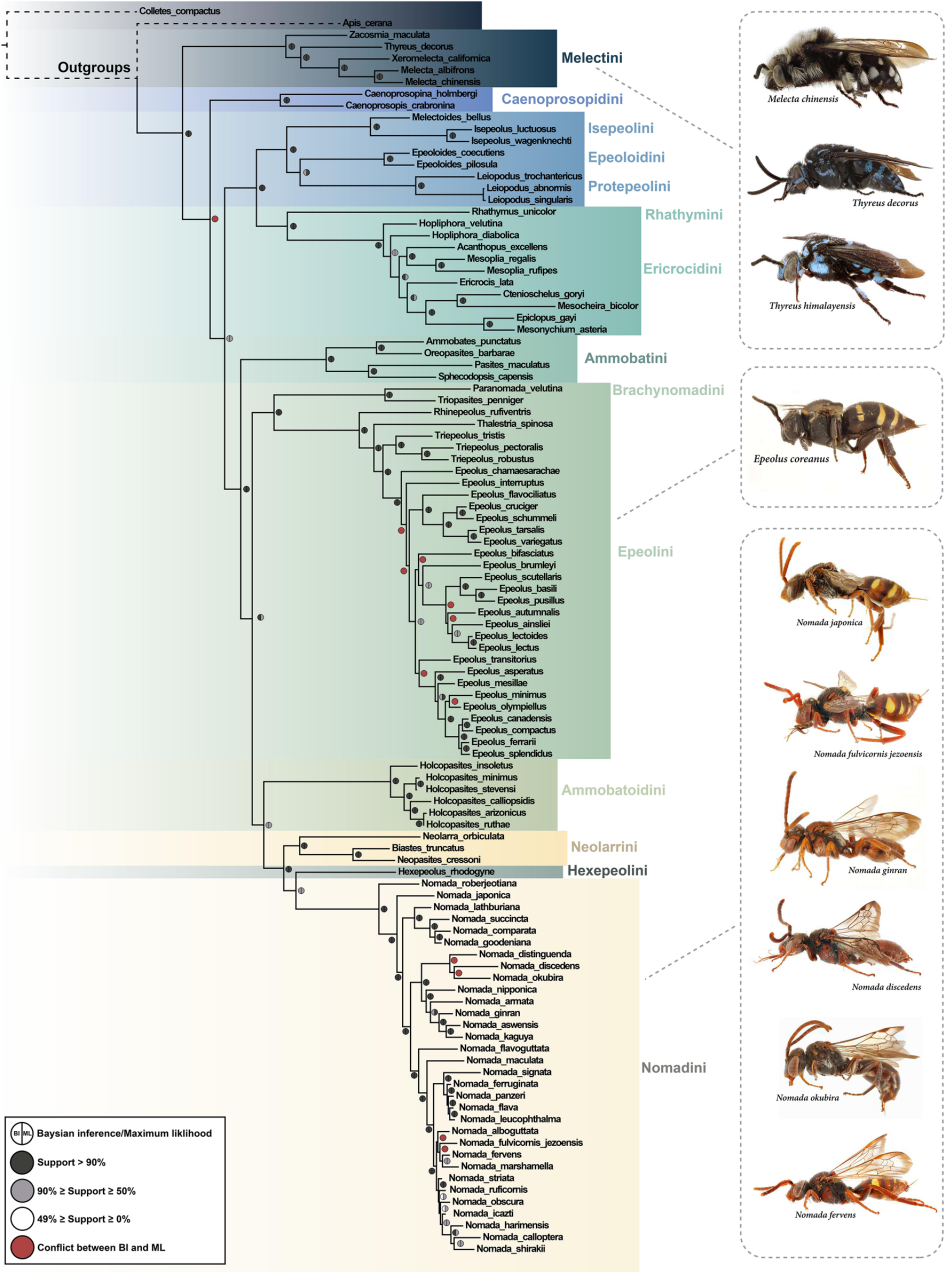
Combined Phylogenetic tree of Nomadinae. Produced with MrBayes. Colors of circles on the node indicate bootstrap supporting values, and the one topological difference between BI and ML trees is presented as a red circle. The tribal classification followed^31^. Habitus images of cuckoo bees was conducted by the first author, Kayun Lim.

Regarding taxon sampling, we targeted species that have both host and body size information without bias. In total, 106 species including 2 species for the outgroup and 104 species for the ingroup were used. We extracted novel sequences from 35 species, and 71 species were obtained from NCBI (Table S1). About 30% of ingroup taxa belong to *Nomada*, which has an relatively unstable phylogenetic position and possesses the most host information in the subfamily. The dataset used for phylogenetic reconstruction contained a total of 4590 bp (657 bp of COI, 709 bp of Ef1α, 1463 bp of Nak, 459 bp of Opsin, 843 bp of PolII, 459 bp of Wng). Phylogenies obtained through Bayesian inference (BI) from MrBayes and maximum likelihood (ML) from IQtree indicate strong support for the monophyly of Nomadinae (Bayesian posterior probability, PP = 100, Maximum likelihood bootstrap values, BS = 99) (Fig. 1).

Melectini was recovered sister to the remaining cleptoparasitic lineages with high support (PP = 100, BS = 98) (Fig. 1). Each of the tribes Caenoprosopidini, Ammobatini, Ammobatoidini, Hexepeolini represented independent clades. We found support for the relationship among the tribes Isepeolini + Epeoloidini (sensu Sless et al., 2022) + Protepeolini (PP = 100, BS = 100,) + Rhathymini + Ericrocidini (PP = 100, BS = 100), and Brachynomadini + Epeolini (PP = 100, BS = 94). The BI and ML trees showed largely congruent topologies except for a few nodes (Fig. 1). Topological differences between BI and ML analyses were found in the tribes Caenoprosopidini, Epeolini, and Nomadini. The tribe Caenoprosopidini was placed sister to Isepeolini + Epeoloidini + Protepeolini in BI, while it was sister to Ammobatini in ML.

The tribe Epeolini formed a distinct monophyletic clade (PP = 100, BS = 100), but some differences between analyses existed within the group. For example, in BI, *Epeolus bifasciatus* formed a subclade with *E. brumleyi*, *E. scutellaris*, *E. basili*, *E. pusillus*, *E. autumnalis*, *E. ainsliei*, *E. lectoides*, and *E. lectus*. However, *E. bifasciatus* grouped with *E. interruptus* in ML. *Epeolus transitorius* was sister to a subclade including *E. asperatus* in BI, but the species was recovered closer to *E. flavociliatus* in ML. *Epeolus chamaesarachae* was recovered as sister to remaining subclades in the tribe with high support in BL (PP = 100), but the species was adjacent to the *E. autumnalis* subclade in ML (BS = 88).

The tribe Nomadini was also strongly supported as monophyletic (PP = 100, BS = 100), and it was recovered sister to Hexepeolini (PP = 78, BS = 94) (Fig. 1). In comparison to the tribal relationships, the infra-tribal relationships of Nomadini were more complex. Some simple cases where single species represented species-groups could be valid, for example the *roberjeotiana* species-group (*N. roberjeotiana*) and *basalis* species-group (*N. japonica*) (sensu Alexander & Schwarz, 1994^41^). Similarly, three members of the *furva* group, *N. distinguenda*, *N. discedens*, *N. okubira*, were supported as monophyletic with strong support (PP = 100, BS = 100). However, the *ruficornis* species-group was more problematic. For example, *N. lathburiana*, which was previously designated as the *ruficornis* species-group^41^, was part of the *bifasciata* species-group. Also, *N. aswensis* which is supposed to be part of the *ruficornis* species-group was part of a clade with *N. nipponica* (*tripsona* species-group), *N. armata*, and *N. ginran* (*armata* species-group). A difference between BI and ML was found in the placement of *N. alboguttata*. In BI, the species formed subclade with *N. fulvicornis jezoensis*, *N. fervens*, and *N. marshamella*. On the other hand, it grouped only with *N. fulvicornis jezoensis* according to ML.

### Ancestral state reconstruction of host use

**Figure 2:**
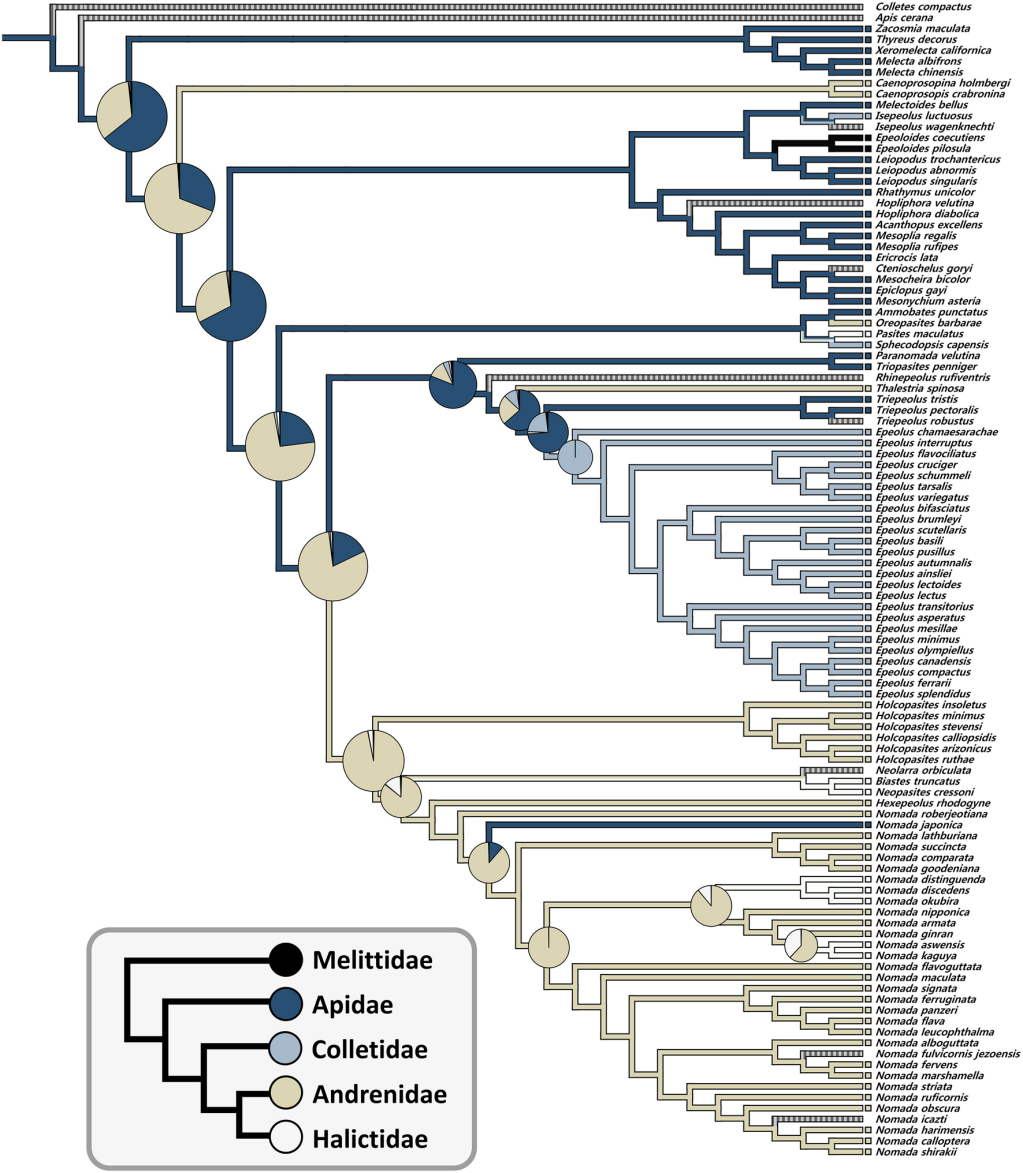
Ancestral state reconstruction of nomadine host associations at the family-level. Produced using the Bayesian analysis. The pie charts represent mean posterior probabilities assessed under RJ-MCMC analysis using Bayestraits. Branch colors indicate the result of parsimony ancestral state reconstruction performed by Mesquite (Black: Melittidae; Navy: Apidae; Sky blue: Colletidae; Ivory: Andrenidae; White: Halictidae; Mixed black/white lines: outgroups or unknown hosts).

The ancestral states of host use in Nomadinae were analyzed at the family level to enable easier interpretation of the results, avoid an overestimation of shifts, and accommodate program analytical limits. Except for a few nodes, parsimony and RJ-MCMC analyses were largely congruent. According to the Bayestraits analysis, the common ancestral host of subfamily Nomadinae was the family Apidae, with a probability of ~ 63% (Fig. 2). This host family is also inferred as the ancestral host for multiple lineages in Nomadinae. Additionally, we found that the host use of Apidae by Melectini, Ericrocidini, Ammobatoidini was conserved. On the other hand, frequent reversal host shifts between Apidae and Andrenidae were detected. After the first reversal host transition from Andrenidae to Apidae happened in the tribal combination of Isepeolini + Parepeolini + Protepeolini + Rathymini + Ericrocidini, host switching also occurred from Apidae to Colletidae and Melittidae (Fig. 2). The common ancestral host of Epeolini was reconstructed as Family Apidae with a probability of ~ 62%. However, a host switch to the family Colletidae in the genus *Epeolus* was observed with a probability of > 99%.

In the clade of Ammobatoidini + Neolarrini + Hexepeolini + Nomadini, their common ancestral host use was yielded as Family Andrenidae with a probability of > 96% (Fig. 2). Thereafter, a host shift occurred from Andrenidae to Halictidae at least three times in this group. To be specific, the first transition happened in the tribe Biasitini, and the remaining two transitions in different clades of the tribe Nomadini. In the tribe Nomadini, there was one switch from Andrenidae to Apidae within our sampling.

The most frequent host switches were observed in the tribe Ammobatini (Fig. 2), and the tribe showed a tendency of reversal host shifts from Andrenidae to Apidae. However, the ancestral host family reconstruction for the tribe was ambiguous and recovered associations with multiple families.

### Allometry and ancestral state reconstruction of body size

The body length of the Nomadinae was on average 9.08 ± 0.36 (mean ± S.E.), widely ranging from 2.5 to 23 mm, and the intertegular distance (ITD) was 2.06 ± 0.11, ranging from 0.48 to 6.82 mm. The mean body length of the hosts was 10.66 ± 0.38, ranging from 3.6 to 23.28 mm and host ITD was 2.84 ± 0.17, ranging from 0.73 to 8.15 mm (see Supplementary Data 1). Our linear regression analysis strongly suggests that the size between Nomadinae and its hosts is highly related in terms of both body length (R^2^ = 0.6879, P < 0.05) and ITD (R^2^ = 0.7620, P < 0.05).

**Figure 3:**
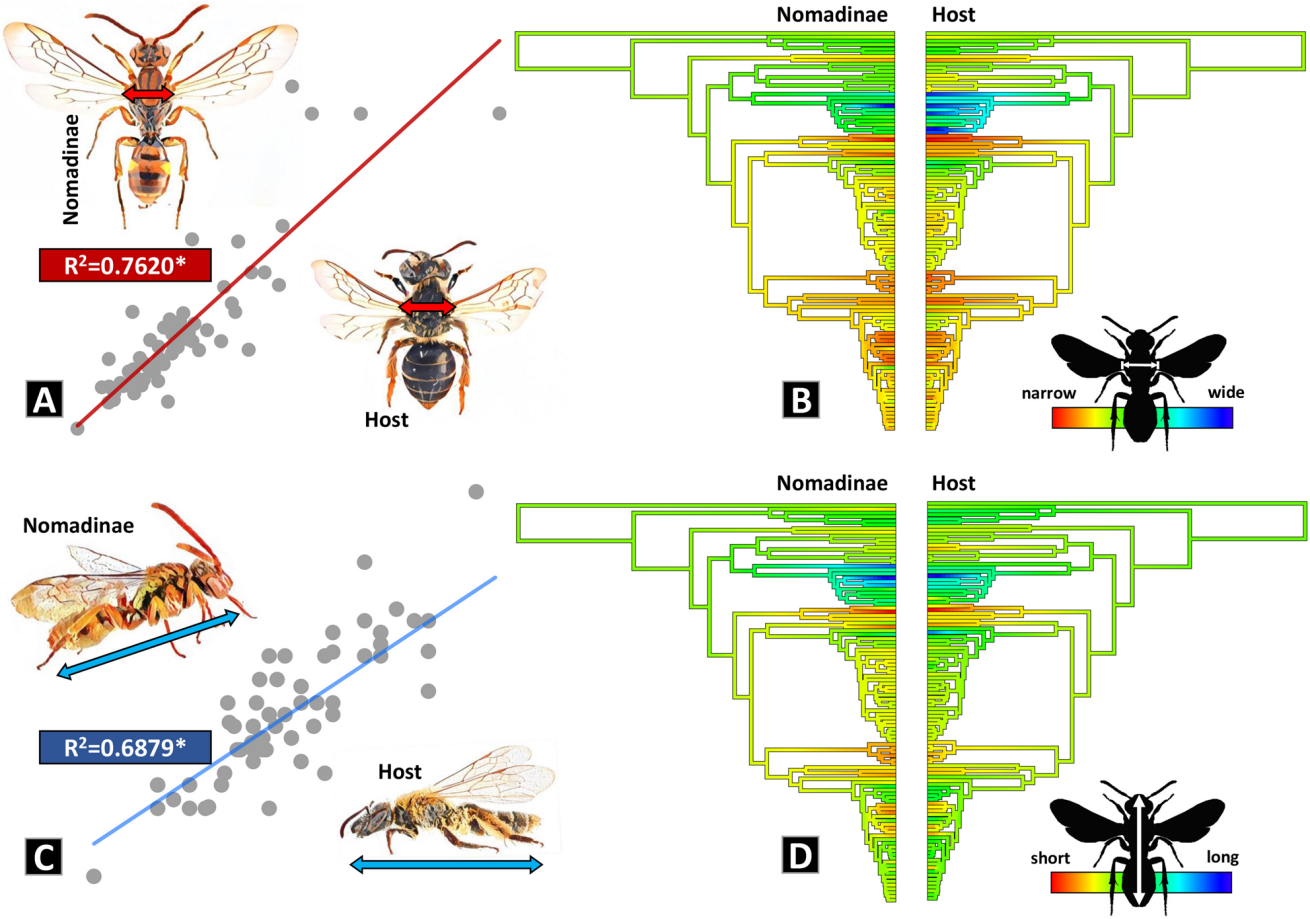
Size correlation between cuckoo bees and hosts. (**A**) Linear regression analysis of ITD (R^2^ = 0.7620, P < 0.05). (**B**) Ancestral character estimation of body width (ITD) of cuckoo bees (left) in accordance with size of the hosts (right). (**C**) Linear regression analysis of entire body length (R^2^ = 0.6879, P < 0.05). (**D**) Ancestral character estimation of body length of cuckoo bees (left) in accordance with size of the hosts (right). Illustrations in (**A**,**C**) were converted from photographs obtained by the first author using PRISMA mobile application.

The ancestral state of ITD and body length was of moderate length (mean of full body length of Melectini: 11.06 mm, median ITD of Melectini: 3 mm; mean full body length of hosts: 13.43 mm; mean ITD of Melectini: 3.59 mm), and the length and width of body forms have evolved from medium to extremes (Fig. 3). It was observed in the tribe Ericrocidini that the body evolved to become longer and wider, but this phenomenon was found only in the tribe Ericrocidini. On the other hand, becoming shorter and narrower was observed in multiple lineages. This distinctly recognizable pattern of shrinking was detected in the tribes Ammobatini, Brachynomadini, Ammobatoidini, Neolarrini, and Nomadini.

Intriguingly, we found potential indicators that changing size is related to host shifting across the phylogeny. To be specific, a considerable body size increase was detected in the tribe Ericrocidini as the host changes from Andrenidae to Apidae. In contrast, both body length and width remained relatively similar within the genus *Epeolus*, which is parasitic on just the genus *Colletes*. This was not always the case, however, as seen among the species-groups of *Nomada*. For example, *N. flavoguttata*, which belongs to the *Nomada ruficornis* species-group and parasitizes the genus *Andrena*, showed a similar body size with species of the *Nomada furva* species-group even though that species-group specializes on a different genus, *Lasioglossum*.

## Discussion

Most of the relationships recovered in this study correspond with a prior investigation that used phylogenomic sampling^31^. Major differences from prior studies include the positioning of the tribes Caenoprosopidini and Ammobatoidini, and the sister group of Nomadini. Firstly, the tribe Caenoprosopidini is located sister to the tribes Isepeolini + Epeoloidini + Protepeolini in BI in our study, while it was placed in the ‘Nomadinae line’ in ML^31^, which includes the Caenoprosopidini, Ammobatini, Ammobatoidini, Brachynomadini, Epeolini, Hexepeolini, Neolarrini, and Nomadini. Second, the tribe Ammobatoidini belonged to a subclade with the tribes Neolarrini, Hexepeolini, and Nomadini in this study. Conversely, it is located closest to the tribes Brachnomadini and Epeolini in previous investigations^31^. Third, the tribe Hexepeolini was inferred as the sister group of the tribe Nomadini. The placement of this tribe has varied even among molecular studies as sometimes it is thought to be sister to the Ammobatoidini, Ammobatini, or Neolarrini^26,28–31^. However, our results are relatively similar to those of the most recent study^31^, where Nomadini was sister to Hexepeolini + Neolarrini. The discrepancy between our results and previous Sanger studies is likely due to differences in taxon sampling, since most prior studies have used relatively few samples. In general, however, we find the most recent Nomadinae phylogenomic study is likely representative^31^, given the depth of data used and their good coverage across taxonomic groups.

Odanaka and colleagues recently conducted the first densely-sampled molecular phylogenetic investigation of *Nomada* using UCE phylogenomic data with 16 species-groups, and their investigation suggested that the *odontophora* species-group is sister to the rest^45^, contrasting with our results that suggested the *roberjeotiana* species-group was sister to the rest. Notably, however, we did not include a sample of the *odontophora* species-group and consequently, here and elsewhere, it seems likely that the differences are due to taxon sampling. Further, difficulties in assigning species-group membership correctly are well known for this genus^40^, such that this could explain some disagreements between our two studies. For example, they recovered the *superba* and *ruficornis* species-groups as paraphyletic, while our study found that the *armata* species-group is paraphyletic, and *ruficornis* species-group is polyphyletic ^45^. Our multi-gene phylogeny supports the designation of *N. ginran* as a member of the *armata* species-group as suggested by^42^. On the other hand, in disagreement with Alexander and Schwarz^41^, *N. aswensis* and *N. kaguya* (previously treated in the *ruficornis* species-group) and *N. nipponica* (previously *tripsona* species-group) were also recovered in the *armata* group. Additionally, *N. lathburiana* (of the *ruficornis* species-group) was recovered in the same clade with *N. succincta*, *N. comparata*, *N. goodeniana*, which belong to the members of the *bifasciata* species-group. Therefore, further investigation with increased taxon sampling is necessary to resolve the true composition and relationships of the *Nomada* species-groups. Again, differences in results between our study and the most recent investigation may be due to differences in taxon sampling, since we primarily focused on species with valid host information. In addition, Odanaka et al. examined *Nomada* phylogeny at a deeper level by using the UCE phylogenomic data with a much denser sampling pool^45^. Given that the phylogenomic approach is possible to provide dept and resolution to the data, the incongruencies between the latest study and the present work are presumably due to the different research methodologies.

Our results largely agree with prior studies, with the family Apidae as an original host and multiple switches to other groups and sometimes back to Apidae. In this way, nomadines appear to initially follow Emery’s rule, with parasites only attacking close relatives as seen with Melectini attacking Anthophorinae, but later with many groups attacking entirely different families very successfully^31^.

In comparision to the most recent reconstruction of Nomadinae^31^, our study determined that host shifting from Apidae to Andrenidae in Nomadinae occurs much earlier. This is because the tribe Caenoprosopidini is shown as sister to the Isepeolini + Epeoloidini + Protepeolini + Rhathymini + Ericrocidini subclade which belongs to the Ericrocidini line (sensu Sless et al., 2022) in our investigation. Conversely, in the previous study, Caenoprosopidini was placed within the Nomadinae line^31^. Denser sampling of these groups will help resolve these and other disagreements.

Host specificity to certain groups has been frequently used to define taxonomic units, including in cuckoo wasps, fig wasps, and some mites^24,49–51^. Some similar examples exist in Nomadinae. For example, the host genus of *Epeolus* is well known to be limited to the genus *Colletes*^52^, and Ericrocidini is limited to Centridini^39^. The common feature of these two examples is that their hosts have specialized nesting strategies, and this may explain some instances of conservatism. Most colletid females apply a cellophane-like layer with their short, bilobed glossa in brood cells^39,53^. Conversely, many hosts of the tribe Ericrocidini collect floral oil for their brood-cell construction^20^. Applying floral oil and other secretions may make it difficult for parasites to access these nests^54^. Both of these specialized nesting behaviors, especially oils which may be hard to digest, could physically inhibit the offspring of parasites^55^. It may be that the adaptations for using these nests, in turn, make it harder for them to switch to other host groups with less specialized behaviors, further enforcing patterns of conservatism^56^.

Of course, exceptions to conservatism of host use exist, such as the use of many different hosts by *Nomada*. Coincidently, this group also represents another inconsistency with the most recent study^31^. We found three host shifts in the tribe, while they found one. There are over 800 species of *Nomada*^35^ and many of them have unknown hosts, so this result depends heavily on taxon sampling. With greater sampling, we would expect additional host shifts. For instance, the host of the *Nomada emarginata* Morawitz (*ruficornis* species-group) is recorded as *Melitta haemorrhoidalis* Fabricus^57^, which belongs to the family Melittidae, but it was not included in our analysis. Similarly, the unused *Nomada articulata* Smith (*erigeronis* species-group) takes *Agapostemon sericeus* Foster and *A. virescens Fabricius* as their hosts^58^, and this could add another shift. As there are numerous species with unknown or unconfirmed hosts, many of which might invoke additional host shifts when added to the phylogeny, it is difficult to estimate the expected total number of host shifts for this group at this time.

Our size allometry analysis strongly supports the positive correlation between parasites and hosts, commonly referred to as Harrison’s rule (HR ^9^;). Further, we found multiple instances where host shifts were linked with body size changes. It is likely that denser taxon sampling will reveal more such linkages between shifts in host use and body size, and it may be that this is a broader co-evolutionary pattern in Nomadinae.

Similar to prior studies on other taxa^59–62^, the body size of cleptoparasitic bees was typically smaller than their hosts in this study. We postulate that there is a “lock-and-key” relationship between the Nomadinae and their hosts (referred to in lice prior, based on the need to fit well on hair ^13^;). Most of the hosts of Nomadinae are soil-nesting, solitary bees. Females make their nests underground by digging, and their nest tunnels are typically just large enough to allow them passage. Because cleptoparasitic bees must enter nests to lay their eggs, they are constrained by the size of these tunnels, nest cells, and even the amount of food provisioned for offspring^25,63,64^. Our investigation supports this idea, with host shifts or stability seemingly influencing body size. For example, as shown in Fig. 3, there is relatively little size fluctuation in the genus *Epeolus*, all of which attack *Colletes*^52^. On the other hand, *Nomada*, which parasitizes a vast array of groups, shows scattered size-shifting corresponding with host switches. The ability of cleptoparasitic bees to modulate their size in response to hosts may contribute to the ability of these bees to switch hosts more readily, which could in turn enable adaptive radiations generated from switching to many new hosts (a possible component of the success of the huge group *Nomada*). Of course, plasticity in host recognition via visual and chemical pathways, as well as adaptability to different nectar and pollen resources, would also be important components.

Another example that highlights the relationship between host switching and body size modulation is found in the tribe Ammobatini. Even though the genus *Melanempis* was excluded from our study because its host is unknown, it is one of the largest nomadines and warrants discussion. According to the most recent study^31^, Ammobatini includes the small-sized *Oreopasites* (as small as 2.2 mm) and *Melanempis* (up to 22 mm), demonstrating huge size variation in the group^36,65^. Alongside this wide range of body sizes, they also parasitize many families: Apidae, Andrenidae, Colletidae, and Halictidae (See supplementary data in^31^). With further data from this group on host associations and body size, and greater taxon sampling, it could be an ideal finer-scale study for looking at the influence of host switching on body size.

Body size is thought to be one of the factors that could contribute to cophylogenetic patterns between hosts and parasites^66^. Food resource requirements being similar among close relatives can help explain such patterns, as they should be relatively similar^13^. Indeed, the ancestral host of Nomadinae was revealed as Apidae in this study (specifically Anthophorinae ^31^,), so co-evolution may have taken place more closely early in the evolution of the nomadines. However, behavioral flexibility can empower parasites to invade novel hosts^56,67^. Such potentials are enhanced when alternative hosts have similar-sized nests^25^, or when their increased abundance makes it more likely for them to be encountered, especially if in the same microhabitat^68^. Host chemical cues also likely play an important role but are relatively poorly understood^69^. Such factors may help explain how single species can exploit many varied hosts^70^. For example, *Nomada flavoguttata* parasites various *Andrena* species such as *Andrena falsifica*, *A. minutula*, *A. minutuloides*, *A. semilaevis*, and *A. subopaca*^35^. Notably, all these host species belong to the same subgenus, *Micrandrena*, which is well-known to have diminutive body size, and may also have similar chemical cues and metabolic requirements^71^. However, occasionally, a single *Nomada* species exploits multiple genera or even families as hosts, such as in *N. imbricata*, parasitizing *Andrena* and *Halictus*^70^. With greater emphasis on natural history studies to make more host-parasite associations, even more disparate host uses might be discovered.

With the combination of our multi-locus phylogeny and allometric data, our understanding of the evolutionary relationship of size fluctuation between cleptoparasitic bees and their hosts has greatly improved. Based on the scenarios and examples discussed above, host switches appear closely related to changes in body size. The ability to adapt to new hosts more quickly in this way could increase speciation rate in the presence of potential new hosts, explaining highly diverse groups like *Nomada*. Ultimately, we confirm Harrison’s rule, the positive correlation of body size between cleptoparasitic bees and hosts, and with further sampling we expect to find an even stronger impact of body size on the evolution of this group.

## Methods

### Taxon sampling

In total, we used 104 species for the ingroup. For the outgroup, *Apis cerana* and *Colletes compactus* were selected to represent a range of relatedness and performed well in pilot analyses, compared to the relationships recovered in a recent phylogenomic analysis^31^. Sequences for 71 species from published papers were taken from NCBI, and we newly added 35 species that have not been analyzed in previous phylogenetic investigations (Table S1). To minimize missing data and ensure better resolution in the phylogenetic tree, we used sequence data only if the number of available genes was greater than or equal to three. The aim of the sampling was to use species that have both host and body size information without bias. When species did not fulfill this condition, they were excluded from this study. However, we made exceptions where necessary to ensure that the resultant phylogenetic reconstructions were reliable, with reference to prior works informing our choices^31,72^.

We placed special focus on sampling the genus *Nomada* as it has highly-diverse host records^31^. Specifically, we expanded the sampling of the *furva* species-group because they are distinctly small-bodied and the parasite of genus *Lasioglossum* (Hymenoptera: Halictidae). Two species, *N. aswensis*, and *N. kaguya*, which belong to the *ruficornis* species-group but show similar biology with the *furva* species-group, were also included in this study. We included two type species, *N. ruficornis* from *ruficornis* species-group and *Nomada armata* from *armata* species-group, to fully investigate the composition of these species-group concepts by Alexander^40^, although greater sampling is necessary to fully define the species-groups^45^. The study used DNA samples of Finnish *Nomada* species from the Genomic Resources collection (https://laji.fi/en/theme/luomusgrc/instructions) of the Finnish Museum of Natural History Luomus (https://www.luomus.fi/en), and the HTTP-URIs of the DNA samples are listed in Table S1. In total, our sampling of the genus *Nomada* represents seven of the 16 species-groups, including a range of host information in three families representing Apidae, Andrenidae, and Halictidae. We could not represent all family-level hosts for this group because there are few species attacking some groups, such as Melittidae, and they were more difficult to sample.

### DNA extraction, PCR amplification, and sequencing

Total genomic DNA was extracted by grinding up detached midlegs or heads of either alcohol vouchers or dried specimens; forceps used for extraction were sanitized before by flame and rinsed in 99% EtOH between each specimen. DNeasy Blood and Tissue kit (QIAGEN, Inc.) was used for the DNA extraction according to the manufacturer’s protocols and stored in − 20 °C. DNA vouchers were deposited in the Insect Biosystematics Laboratory, Seoul National University.

Genes commonly used in Anthophila phylogenetics were selected. One mitochondrial protein coding gene, cytochrome oxidase subunit I gene (COI)^52,73^, and five nuclear protein-coding genes (EF-1α, long-wavelength rhodopsin (opsin), NaK, pol II, and wingless) that were used in previously published literature^28,29,52,55,74–78^ were chosen for this study. In some cases, COI from NCBI were used.

PCR products were amplified using Accupowder PCR Premix (Bioneer, Daejeon, Korea) in a 20 µl reaction mixture. In case DNA extraction was needed from bad quality specimens, we redesigned the partial primers in threefold. The primers used in this study and specific PCR conditions are listed in Table S2. After the amplification process, PCR products were purified and sequenced at Bionics CO. (Seoul, Korea).

Using the SeqMan Pro version 7.1.0., raw sequence data were assembled, checked, and trimmed. Sequence alignment of six genes were performed in MAFFT version 7 (https://mafft.cbrc.jp/alignment/server/)^79^. All the sequences were adjusted in Mega 7 with the amino acid translation option. The aligned sequences were combined using SequenceMatrix Windows ver. 1.8^80^.

### Phylogenetic analyses

Phylogenetic analyses were performed using both Bayesian inference (BI) and Maximum likelihood (ML). The Bayesian inference analysis was conducted with MrBayes 3.2.7a^81^. The best substitution model for BI was selected for each partition under the Bayesian Information Criterion (BIC) using ModelFinder in IQTREE and the same protocol was used in searching for the best substitution model for ML^82,83^. GTR + F + I + G4 for COI, TIMe + I + G4 for ef1α, TIM3 + F + I + G4 for Nak, TIM3e + I + G4 for Opsin, TIM2 + F + I + G4 for PolII, and TIM + F + I + G4 for Wng were selected as the best fitting models for BI. For ML, GTR + F + I + G4 for COI, TIM + F + I + G4 for ef1α, TIM3 + F + I + G4 for Nak, TIM3e + I + G4 for Opsin, TIM2 + F + I + G4 for PolII, TIM + F + I + G4 for Wng were selected^84^. However, since TIM models cannot be used in MrBayes, GTR + I + G was used to run the BI analysis except COI. For the MrBayes analysis, we ran 20 million Markov chain Monte Carlo (MCMC) generations and trees were sampled every 100 generations. We ran one cold chain and three heated chains for each MCMC analysis. The first two million sampled trees were discarded as burn-in. Branch support for the maximum likelihood analysis was computed using the UltraFast bootstrap approximation (UBS ^85^;) with 1,000 replicates.

### Size allometry

We measured two traits of the Nomadinae and their hosts. One is the intertegular distance (minimum distance between the tegulae), which is a useful estimator for the size of bees^86^. The other is total body length, which was measured as the maximum length distance from the head to end of the final tergite exclusive of exserted stingers or genitalia. (if curled, this was accounted for using a multi-stage measurement). In total, we obtained about 1,300 body size data from Nomadinae and their hosts (Body length: 73; ITD: 65 out of 106 species of Nomadinae and their corresponding hosts). Among them, we used specimens of 24 species (host: 7, Nomadinae: 17) and prepared photographs using the Microscope (DM 4000B, Leica Microsystem, Wetzlar, Germany) with a USB digital camera (Infinity3, Lumenera Corporation, Ottawa, Ontario) and measured the value of the traits using the measurement option. Since there can be a size difference between male and female bees, measurements were only taken from females. We also collected data from literature such as taxonomic papers, online-accessible specimen photographs in museums, or from taxonomists (see Supplementary Data 1). The number of measured individuals was different per species because we gathered the data from various sources. Therefore, we used the average value of measurements for the data analysis. Linear regressions were conducted using SPSS Statistics 25 (IBM, Armonk, NY, USA) to gauge the dependence of nomadine body length on host body length, as well as nomadine ITD on host ITD.

### Ancestral character state reconstruction

The host information of each nomadine species was extracted from the literature (See supplementary data 1). Where multiple species were reported as hosts, the data was prioritized when the parasitic larvae were found in their nests or when direct intrusion of the parasite to the host nests was observed. However, when such confirmation was absent, data arose from phenological synchrony between parasites and hosts and information provided by taxonomists, including hypotheses raised in the literature.

The hosts of Nomadinae were coded as five discrete states by families: (A) Andrenidae, (B) Apidae, (C) Colletidae, (D) Halticidae, and (E) Melittidae. This may underestimate the number of shifts because different lineages are used in some families, but as it is difficult to exactly know when switches occurred this is in some cases conservative, and avoids the artificial inflation of the number of shifts. The trace character history function in in Mesquite 3.31 was used to map the evolutionary history of host use on a single tree with parsimony approach^87^ was adopted. The probability of the ancestral state of each node was calculated by Bayestraits v3.0^88^ using reversible jump Markov Chain Monte Carlo (RJ-MCMC). An exponential distribution was implemented, seeding from a uniform prior in an interval of 0–100. We ran 50 million iterations, sampling every 1,000th iteration. The first million iterations were discarded as burn-in. Acceptance rates were automatically adjusted and achieved in the preferred range of near 35%.

To trace the evolutionary history of intertegular distance and body size, we used ancestral state reconstruction using the maximum likelihood (ML) method in the R package ‘Phytools’^89^. The resulting Bayesian tree was converted into a dendrogram, the missing data vector was estimated using ML, and the ContMap function was used to visualize their evolutionary history.

## Supplementary Information

Supplementary Information 1.

Supplementary Information 2.

Supplementary Information 3.

## Data Availability

All raw sequence data have been deposited in NCBI. Accession codes are as follows: OM722151-OM722175; OM850346-OM850368; OM906091-OM906191; OM912457-OM912460.

## References

[1] Fisher DO, Dickman CR (1993). Body size-prey size relationships in insectivorous marsupials: Tests of three hypotheses. Ecology.

[2] Woodward G, Hildrew AG (2002). Body-size determinants of niche overlap and intraguild predation within a complex food web. J. Anim. Ecol..

[3] Duellman, W. E. Cusco Amazónico. *Comstock Publishing Associates*, Cornell Univ. Press, Ithaca, NY 456pp. (2005).

[4] Peters RH (1983). The Ecological Implications of Body Size.

[5] Fisher JT, Anholt B, Volpe JP (2011). Body mass explains characteristic scales of habitat selection in terrestrial mammals. Ecol. Evol..

[6] Wikelski M (2005). Evolution of body size in Galapagos marine iguanas. Proc. R. Soc. B Biol. Sci..

[7] Sibly RM, Brown JH (2007). Effects of body size and lifestyle on evolution of mammal life histories. Proc. Natl. Acad. Sci..

[8] Woodward G (2005). Body size in ecological networks. Trends Ecol. Evol..

[9] Harrison L (1915). Mallophaga from apteryx, and their significance; with a note on the genus *rallicola*. Parasitology.

[10] Harvey, P. H., & Keymer, A. E. Comparing life histories using phylogenies. *Philos. Trans. R. Soc. Lond. Ser. B Biol. Sci*. **332**, 31–39 (1991).

[11] Kirk WD (1991). The size relationship between insects and their hosts. Ecol. Entomol..

[12] Morand S, Legendre P, Gardner SL, Hugot JP (1996). Body size evolution of oxyurid (Nematoda) parasites: The role of hosts. Oecologia.

[13] Morand S, Hafner MS, Page RD, Reed DL (2000). Comparative body size relationships in pocket gophers and their chewing lice. Biol. J. Lin. Soc..

[14] Poulin R, Hamilton WJ (1997). Ecological correlates of body size and egg size in parasitic Ascothoracida and Rhizocephala (Crustacea). Acta Oecol..

[15] Sasal, P. E., Trouve ´, S., Müller-Graf, C. & Morand, S. Specificity and host predictability: A comparative analysis amongmonogenean parasites of fish. *J. Anim. Ecol*. **68**, 437–444 (1999).

[16] Gould GC, MacFadden BJ (2004). Gigantism, dwarfism, and Cope's rule:“Nothing in evolution makes sense without a phylogeny”. Bull. Am. Mus. Nat. Hist..

[17] Nagler C (2017). The bigger, the better? Volume measurements of parasites and hosts: Parasitic barnacles (Cirripedia, Rhizocephala) and their decapod hosts. PLoS ONE.

[18] Harnos A (2017). Size matters for lice on birds: Coevolutionary allometry of host and parasite body size. Evolution.

[19] Maestri R (2020). Harrison's rule scales up to entire parasite assemblages but is determined by environmental factors. J. Anim. Ecol..

[20] Danforth BN, Minckley RL, Neff JL (2019). The Solitary Bees.

[21] Rozen JG (1965). Biological notes on the cuckoo bee genera *Holcopasites* and *Neolarra* (Hymenoptera: Apoidea). J. New York Entomol. Soc..

[22] Wcislo WT, Cane JH (1996). Floral resource utilization by solitary bees (Hymenoptera: Apoidea) and exploitation of their stored foods by natural enemies. Annu. Rev. Entomol..

[23] Rozen JG (2003). Eggs, ovariole numbers, and modes of parasitism of cleptoparasitic bees, with emphasis on Neotropical species (Hymenoptera: Apoidea). Am. Mus. Novit..

[24] Alexander BA (1990). A cladistic analysis of the nomadine bees (Hymenoptera: Apoidea). Syst. Entomol..

[25] Habermannová J, Bogusch P, Straka J (2013). Flexible host choice and common host switches in the evolution of generalist and specialist cuckoo bees (Anthophila: Sphecodes). PLoS ONE.

[26] Bossert S (2019). Combining transcriptomes and ultraconserved elements to illuminate the phylogeny of Apidae. Mol. Phylogenet. Evol..

[27] Ascher, J. S. & Pickering, J. *Discover Life bee species guide and world checklist (Hymenoptera: Apoidea: Anthophila)* http://www.discoverlife.org/mp/20q?guide=Apoidea_species (2021).

[28] Cardinal S, Straka J, Danforth BN (2010). Comprehensive phylogeny of apid bees reveals the evolutionary origins and antiquity of cleptoparasitism. Proc. Natl. Acad. Sci..

[29] Litman JR, Praz CJ, Danforth BN, Griswold TL, Cardinal S (2013). Origins, evolution, and diversification of cleptoparasitic lineages in long-tongued bees. Evolution.

[30] Martins AC, Luz DR, Melo GA (2018). Palaeocene origin of the Neotropical lineage of cleptoparasitic bees Ericrocidini-Rhathymini (Hymenoptera, Apidae). Syst. Entomol..

[31] Sless TJ (2022). Phylogenetic relationships and the evolution of host preferences in the largest clade of brood parasitic bees (Apidae: Nomadinae). Mol. Phylogenet. Evol..

[32] Maeta, Y., Goukon, K., Sugiura, N., & Miyanaga, R. Host records of cleptoparasitic bees in Japan (Hymenoptera, Apoidea). *昆蟲*, **64**, 830–842 (1996).

[33] Amiet F, Herrmann M, Müller A, Neumeyer R (2007). Apidae 5: Ammobates. Ammobatoides, Anthophora, Biastes, Ceratina, Dasypoda, Epeoloides, Epeolus, Eucera, Macropis, Melecta, Melitta, Nomada, Pasites, Tetralonia, Thyreus, Xylocopa Centre suisse de cartographie de la faune..

[34] Onuferko TM (2018). A revision of the cleptoparasitic bee genus *Epeolus* Latreille for Nearctic species, north of Mexico (Hymenoptera, Apidae). ZooKeys.

[35] Smit J (2018). Identification key to the European species of the bee genus *Nomada* Scopoli, 1770 (Hymenoptera: Apidae), including 23 new species. Entomofauna..

[36] Rozen Jr, J. G. Systematics and host relationships of the cuckoo bee genus *Oreopasites* (Hymenoptera, Anthophoridae, Nomadinae). *American Museum novitates*. no. 3046, 56pp (1992).

[37] Rozen JG (2019). Early nesting biology of the bee *Caupolicana yarrowi* (Cresson) (Colletidae: Diphaglossinae) and its cleptoparasite *Triepeolus grandis* (Friese) (Apidae: Nomadinae). Am. Mus. Novit..

[38] Orr MC, Griswold TL (2015). A review of the cleptoparasitic bee genus *Townsendiella* (Apidae, Nomadinae, Townsendiellini), with the description of a new species from Pinnacles National Park. ZooKeys.

[39] Michener CD (2007). The Bees of the World.

[40] Alexander BA (1994). Species-Groups and Cladisitic Analysis of the Cleptoparasitic Bee Genus *Nomada* (Hymenoptera: Apoidea). Univ. Kansas Sci. Bull..

[41] Alexander, B. A., & Schwarz, M. A catalog of the species of *Nomada* (Hymenoptera: Apoidea) of the world. *Univ. Kansas Sci. Bull*. **55**, 239–270 (1994).

[42] Mitai K, Tadauchi O (2007). Taxonomic study of the Japanese species of the *Nomada ruficornis* species group (Hymenoptera, Apidae) with remarks on Japanese fauna of the genus *Nomada*. Esakia.

[43] Proshchalykin MY, Lelej AS (2010). Review of the *Nomada roberjeotiana* species-group (Hymenoptera: Apidae) of Russia, with description of new species. Zootaxa.

[44] Won, H. S. Systematics of the genus *Nomada* (Hymenoptera: Apidae) in Korea. Ph.D. thesis. 1–132 (2006).

[45] Odanaka KA, Branstetter MG, Tobin KB, Rehan SM (2022). Phylogenomics and historical biogeography of the cleptoparasitic bee genus Nomada (Hymenoptera: Apidae) using ultraconserved elements. Mol. Phylogenet. Evol..

[46] Bossert S (2021). Phylogeny, biogeography and diversification of the mining bee family Andrenidae. Syst. Entomol..

[47] Schrottky, C. *Ensaio sobre as abelhas solitarias do Brazil* (Revista do Museo Paulista, 1902).

[48] Emery C (1909). Über den Ursprung der dulotischen, parasitischen und myrmekophilen Ameisen. Biol. Cent..

[49] Costa, M. *Cerambylaelaps nadchatrami*, n. gen, n. sp., an unusual Mesostigmatic mite (Acari) associated with a cerambycid beetle in Malaysia. *Acarologia*. **20**, 188–195 (1979).

[50] Kimsey LS (1992). Functional morphology of the abdomen and phylogeny of the chrysidid wasps (Hymenoptera: Chrysididae). J. Hymenop. Res..

[51] Chen L (2021). Adaptation of Fig Wasps (Agaodinae) to their host revealed by large-scale transcriptomic data. Insects..

[52] Onuferko TM, Bogusch P, Ferrari RR, Packer L (2019). Phylogeny and biogeography of the cleptoparasitic bee genus *Epeolus* (Hymenoptera: Apidae) and cophylogenetic analysis with its host bee genus *Colletes* (Hymenoptera: Colletidae). Mol. Phylogenet. Evol..

[53] Almeida EA (2008). Colletidae nesting biology (Hymenoptera: Apoidea). Apidologie.

[54] Alves-dos-Santos I, Melo GA, Rozen JG (2002). Biology and immature stages of the bee tribe Tetrapediini (Hymenoptera: Apidae). Am. Mus. Novit..

[55] Policarová J, Cardinal S, Martins AC, Straka J (2019). The role of floral oils in the evolution of apid bees (Hymenoptera: Apidae). Biol. J. Lin. Soc..

[56] Bush SE (2009). Does behavioural flexibility facilitate host switching by parasites?. Funct. Ecol..

[57] Westrich, P. The wild bees of Baden-Württemberg (Stuttgard: Eugen Ulmer) (1989).

[58] Snelling, R. R. Contributions toward a revision of the New World nomadine bees. A partitioning of the genus *Nomada* (Hymenoptera: Anthophoridae). *Natl. Hist. Museum Los Angeles County*. **376**, 1–32 (1986).

[59] Price P (1980). The Evolutionary Biology of Parasites.

[60] Hanken J, Wake DB (1993). Miniaturization of body size: organismal consequences and evolutionary significance. Annu. Rev. Ecol. Syst..

[61] Tsai, M. L., Li, J. J. & Dai, C. F. How host size may constrain the evolution of parasite body size and clutch size. The parasitic isopod *Ichthyoxenus fushanensis* and its host fish, *Varicorhinus bacbatulus*, as an example. *Oikos*. **92**, 13–19 (2001).

[62] Lafferty KD, Kuris AM (2002). Trophic strategies, animal diversity and body size. Trends Ecol. Evol..

[63] Roulston TAH, Cane JH (2000). The effect of diet breadth and nesting ecology on body size variation in bees (Apiformes). J. Kansas Entomol. Soc..

[64] Radmacher S, Strohm E (2010). Factors affecting offspring body size in the solitary bee *Osmia bicornis* (Hymenoptera, Megachilidae). Apidologie.

[65] Pauly, A *et al. Hymenoptera Apoidea de Madagascar et des îles voisines*. (Koninklijk Museum voor Midden-Afrika, 2001).

[66] Sweet AD, Wilson RE, Sonsthagen SA, Johnson KP (2020). Lousy grouse: Comparing evolutionary patterns in Alaska galliform lice to understand host evolution and host–parasite interactions. Ecol. Evol..

[67] Baldwin JM (1896). A new factor in evolution. Am. Nat..

[68] Parker J, Rabeling C (2020). Evolution: Shape-shifting social parasites. Curr. Biol..

[69] Tengö J, Bergström G (1977). Cleptoparasitism and odor mimetism in bees: do *Nomada* males imitate the odor of *Andrena* females?. Science.

[70] Packard AS (1868). The home of the bees (Concluded). Am. Nat..

[71] Dardón MJ, Torres F, Ornosa C (2014). The subgenus *Andrena* (*Micrandrena*) (Hymenoptera: Andrenidae) in the Iberian Peninsula. Zootaxa.

[72] Branstetter MG (2017). Phylogenomic insights into the evolution of stinging wasps and the origins of ants and bees. Curr. Biol..

[73] Hebert, P. D., Cywinska, A., Ball, S. L., & Dewaard, J. R. Biological identifications through DNA barcodes. *Proc. R. Soc. Lond. Ser. B Biol. Sci*. **270**, 313–321 (2003). 10.1098/rspb.2002.2218 PMC1691236 12614582

[74] Ascher JS, Danforth BN, Ji S (2001). Phylogenetic utility of the major opsin in bees (Hymenoptera: Apoidea): A reassessment. Mol. Phylogenet. Evol..

[75] Danforth BN, Sipes S, Fang J, Brady SG (2006). The history of early bee diversification based on five genes plus morphology. Proc. Natl. Acad. Sci..

[76] Diestelhorst O, Lunau K (2008). Beitrag zur Klärung des Artstatus von *Nomada succincta* Panzer, 1798 (Hymenoptera, Apidae). Entomologie heute..

[77] Gerth M, Röthe J, Bleidorn C (2013). Tracing horizontal Wolbachia movements among bees (Anthophila): A combined approach using multilocus sequence typing data and host phylogeny. Mol. Ecol..

[78] Schmidt S, Schmid-Egger C, Morinière J, Haszprunar G, Hebert PD (2015). DNA barcoding largely supports 250 years of classical taxonomy: identifications for Central European bees (Hymenoptera, A poidea partim). Mol. Ecol. Resour..

[79] Katoh K, Standley DM (2013). MAFFT multiple sequence alignment software version 7: Improvements in performance and usability. Mol. Biol. Evol..

[80] Vaidya G, Lohman DJ, Meier R (2011). SequenceMatrix: concatenation software for the fast assembly of multi-gene datasets with character set and codon information. Cladistics.

[81] Ronquist, F. *et al.* MrBayes 3.2: efficient Bayesian phylogenetic inference and model choice across a large model space. *Syst. Biol.*, **61**, 539–542 (2012). 10.1093/sysbio/sys029 PMC3329765 22357727

[82] Kalyaanamoorthy S, Minh BQ, Wong TK, Von Haeseler A, Jermiin LS (2017). ModelFinder: Fast model selection for accurate phylogenetic estimates. Nat. Methods.

[83] Nguyen LT, Schmidt HA, Von Haeseler A, Minh BQ (2015). IQ-TREE: A fast and effective stochastic algorithm for estimating maximum likelihood phylogenies. Mol. Biol. Evol..

[84] Chernomor O, Von Haeseler A, Minh BQ (2016). Terrace aware data structure for phylogenomic inference from supermatrices. Syst. Biol..

[85] Hoang, D.T., Chernomor, O., Haeseler, A.V., Minh, B.Q., & Vinh, L. S. UFBoot2: Improving the ultrafast bootstrap approximation. *Mol. Biol. Evol.* in press (2017). 10.1093/molbev/msx281 PMC5850222 29077904

[86] Cane JH (1987). Estimation of bee size using intertegular span (Apoidea). J. Kansas Entomol. Soc..

[87] Maddison, W. P. & Maddison, D. R. Mesquite: A modular system for evolutionary analysis. Version 3.31 http://mesquiteproject.org (2017).

[88] Meade, A. & Pagel, M. 2017. BayesTraits V3.0. http://www.evolution.rdg.ac.uk/BayesTraitsV3/BayesTraitsV3.html.

[89] Revell LJ (2012). phytools: An R package for phylogenetic comparative biology (and other things). Methods Ecol. Evol..

