# Supplementary material for: Harrison’s rule corroborated for the body size of cleptoparasitic cuckoo bees (Hymenoptera: Apidae: Nomadinae) and their hosts
Source: Sci Rep. 2022 Jun 29;12:10984. doi: 10.1038/s41598-022-14938-9 (PMC9243014; doi:10.1038/s41598-022-14938-9)
Supplement: Supplementary file 2 — Supplementary Information 2. [file 41598_2022_14938_MOESM2_ESM.docx]

**Table S1. List of the Subfamily Nomadinae for this study.**

| Tribe | Species | Host | GeneBank accession number | | | | | | Specimen  Reference |
| --- | --- | --- | --- | --- | --- | --- | --- | --- | --- |
|  |  |  | **COI** | **EF1α** | **Nak** | **Opsin** | **PolⅡ** | **Wingless** |  |
| Outgroup | *Colletes compactus* | - | DQ872684 | DQ884642 | MN367513 | DQ884542 | MN367595 | DQ884794 | 1 |
|  | *Apis cerana* | - | KM242593 | EU184774 | EU184750 | AB355818 | EU184733 | EU184716 | 1 |
| Melectini | *Zacosmia maculata* | B | MN344989 | AY585117 | GU245117 | AF344637 | AY945176 | GU245570 | 1 |
|  | *Thyreus decorus* | B | OM722174 | OM906191 | OM906168 | OM906113 | OM906140 | OM850366 | 2 |
|  | *Xeromelecta californica* | B | MK919652 | GU244955 | GU245116 | AF344613 | GU245407 | GU245569 | 1 |
|  | *Melecta albifrons* | B | MK919610 | GU244998 | GU245120 | HM211837 | GU245409 | GU245573 | 1 |
|  | *Melecta chinensis* | B | OM722175 | - | OM906169 | OM906114 | OM906141 | OM850367 | 2 |
| Caenoprosopidini | *Caenoprosopina holmbergi* | D | MK919570 | GU244983 | GU245194 | GU245325 | GU245476 | GU245650 | 1 |
|  | *Caenoprosopis crabronina* | A | MK919571 | GU245020 | GU245195 | GU245326 | GU245477 | GU245651 | 1 |
| Isepeolini | *Melectoides bellus* | B | MK919611 | GU244999 | GU245115 | HM211836 | GU245406 | GU245568 | 1 |
|  | *Isepeolus luctuosus* | C | KX821019 | GU244953 | GU245113 | GU245277 | GU245404 | GU245566 | 1 |
|  | *Isepeolus wagenknechti* | N.A. | KX821183 | GU244954 | GU245114 | GU245278 | GU245405 | GU245567 | 1 |
| Epeoloidini | *Epeoloides coecutiens* | E | MK919586 | GU244987 | GU245131 | HM211838 | GU245418 | GU245584 | 1 |
|  | *Epeoloides pilosula* | E | JN293445 | GU244966 | GU245129 | GU245287 | GU245416 | GU245582 | 1 |
| Protepeolini | *Leiopodus trochantericus* | B | MK919603 | GU244970 | GU245136 | GU245291 | GU245422 | GU245589 | 1 |
|  | *Leiopodus abnormis* | B | X | GU244969 | GU245135 | GU245290 | GU245421 | GU245588 | 1 |
|  | *Leiopodus singularis* | B | X | AY585113 | GU245133 | AF344624 | AY945137 | GU245586 | 1 |
| Rhathymini | *Rhathymus unicolor* | B | X | GU244973 | GU245139 | GU245294 | GU245425 | GU245592 | 1 |
| Ericrocidini | *Hopliphora velutina* | N.A. | MK919599 | GU244939 | GU245086 | GU245258 | GU245379 | GU245539 | 1 |
|  | *Hopliphora diabolica* | B | KY084893 | KY084881 | X | KY084866 | KY084853 | X | 1 |
|  | *Acanthopus excellens* | A | KY084889 | KY084876 | X | KY084863 | KY084849 | X | 1 |
|  | *Mesoplia regalis* | B | KY084901 | KY084887 | X | KY084871 | KY084859 | X | 1 |
|  | *Mesoplia rufipes* | B | MK919618 | GU244938 | GU245085 | GU245257 | GU245378 | GU245538 | 1 |
|  | *Ericrocis lata* | B | MN345705 | GU244936 | GU245083 | GU245255 | GU245376 | GU245536 | 1 |
|  | *Ctenioschelus goryi* | N.A. | KY084892 | GU244941 | GU245088 | GU245260 | GU245381 | GU245541 | 1 |
|  | *Mesocheira bicolor* | B | MK919616 | GU244940 | GU245087 | GU245259 | GU245380 | GU245540 | 1 |
|  | *Epiclopus gayi* | B | MK919589 | GU244935 | GU245082 | GU245254 | GU245375 | GU245535 | 1 |
|  | *Mesonychium asteria* | B | KY084898 | GU244937 | GU245084 | GU245256 | GU245377 | GU245537 | 1 |
| Ammobatini | *Ammobates punctatus* | B | KJ838996 | GU245011 | GU245179 | HM211841 | GU245463 | GU245635 | 1 |
|  | *Oreopasites barbarae* | A | MK919624 | GU245008 | GU245176 | AF344626 | GU245460 | GU245632 | 1 |
|  | *Pasites maculatus* | D | MN344150 | GU245035 | GU245180 | HM211842 | GU245464 | GU245636 | 1 |
|  | *Sphecodopsis capensis* | C | MK919639 | GU245009 | GU245177 | GU245317 | GU245461 | GU245633 | 1 |
| Brachynomadini | *Paranomada velutina* | B | MK919627 | AY585115 | GU245190 | AF344627 | AY945154 | GU245646 | 1 |
|  | *Triopasites penniger* | B | MK919650 | GU245018 | GU245191 | AF344633 | GU245473 | GU245647 | 1 |
| Epeolini | *Rhinepeolus rufiventris* | N.A. | MK919638 | GU245027 | GU245202 | GU245331 | GU245483 | GU245658 | 1 |
|  | *Thalestria spinosa* | A | MN342317 | GU245024 | GU245199 | GU245328 | GU245480 | GU245655 | 1 |
|  | *Triepeolus tristis* | B | MN342316 | MN367540 | MN367496 | MN367458 | MN367574 | MN367661 | 1 |
|  | *Triepeolus pectoralis* | B | MN343742 | MN367539 | MN367495 | MN367446 | MN367581 | MN367659 | 1 |
|  | *Triepeolus robustus* | N.A. | MK919585 | GU245023 | GU245198 | AF344634 | AY945170 | GU245654 | 1 |
|  | *Epeolus chamaesarachae* | C | MH089974 | MN367522 | MN367484 | MN367461 | MN367573 | MN367638 | 1 |
|  | *Epeolus interruptus* | C | MH089961 | MN367536 | MN367494 | MN367457 | MN367571 | MN367654 | 1 |
|  | *Epeolus flavociliatus* | C | MN342311 | MN367528 | MN367482 | X | MN367580 | MN367658 | 1 |
|  | *Epeolus cruciger* | C | MN342321 | MN367525 | MN367479 | MN367459 | MN367579 | MN367652 | 1 |
|  | *Epeolus schummeli* | C | MN342314 | MN367526 | MN367480 | KC798351 | MN367577 | MN367653 | 1 |
|  | *Epeolus tarsalis* | C | MN342315 | MN367527 | MN367481 | MN367460 | MN367578 | MN367655 | 1 |
|  | *Epeolus variegatus* | C | MK919587 | GU244988 | GU245203 | HM211846 | GU245484 | GU245659 | 1 |
|  | *Epeolus bifasciatus* | C | MH089986 | MN367535 | MN367500 | MN367462 | MN367575 | MN367649 | 1 |
|  | *Epeolus brumleyi* | C | MH089901 | MN367538 | MN367493 | MN367456 | MN367576 | MN367657 | 1 |
|  | *Epeolus scutellaris* | C | HQ552250 | GU245022 | GU245197 | AF344596 | GU245479 | GU245653 | 1 |
|  | *Epeolus basili* | C | MH090001 | MN367532 | MN367497 | MN367444 | MN367557 | MN367636 | 1 |
|  | *Epeolus pusillus* | C | MH089868 | MN367533 | MN367498 | MN367445 | MN367558 | MN367637 | 1 |
|  | *Epeolus autumnalis* | C | MH089931 | MN367534 | MN367499 | MN367441 | MN367562 | MN367640 | 1 |
|  | *Epeolus ainsliei* | C | MH089843 | MN367531 | MN367478 | MN367440 | MN367561 | MN367639 | 1 |
|  | *Epeolus lectoides* | C | MH090009 | MN367529 | MN367476 | MN367442 | MN367560 | MN367641 | 1 |
|  | *Epeolus lectus* | C | MH089993 | MN367530 | MN367477 | MN367443 | MN367559 | MN367642 | 1 |
|  | *Epeolus transitorius* | C | MN342320 | MN367537 | MN367483 | MN367463 | MN367572 | MN367656 | 1 |
|  | *Epeolus asperatus* | C | MH090008 | MN367514 | MN367491 | MN367454 | MN367570 | MN367660 | 1 |
|  | *Epeolus mesillae* | C | MH089889 | MN367515 | MN367490 | MN367455 | MN367569 | MN367651 | 1 |
|  | *Epeolus minimus* | C | MH090003 | MN367518 | MN367492 | MN367452 | MN367568 | MN367646 | 1 |
|  | *Epeolus olympiellus* | C | MH089905 | MN367517 | MN367489 | MN367453 | MN367566 | MN367647 | 1 |
|  | *Epeolus canadensis* | C | MH089848 | MN367516 | MN367485 | MN367451 | MN367567 | MN367643 | 1 |
|  | *Epeolus compactus* | C | MH089882 | MN367521 | MN367487 | MN367450 | MN367565 | MN367645 | 1 |
|  | *Epeolus ferrarii* | C | MH089922 | MN367519 | MN367486 | MN367448 | MN367563 | MN367644 | 1 |
|  | *Epeolus splendidus* | C | MH089954 | MN367520 | MN367488 | MN367449 | MN367564 | MN367648 | 1 |
| Ammobatoidini | *Holcopasites insoletus* | A | X | GU245014 | GU245184 | GU245320 | GU245467 | GU245640 | 1 |
|  | *Holcopasites minimus* | A | X | GU245016 | GU245187 | GU245322 | GU245470 | GU245643 | 1 |
|  | *Holcopasites stevensi* | A | X | GU245015 | GU245186 | GU245321 | GU245469 | GU245642 | 1 |
|  | *Holcopasites calliopsidis* | A | MK919598 | GU245012 | GU245182 | AF344600 | GU245465 | GU245638 | 1 |
|  | *Holcopasites arizonicus* | A | X | GU245013 | GU245183 | GU245319 | GU245466 | GU245639 | 1 |
|  | *Holcopasites ruthae* | A | X | AY585112 | GU245181 | AF344602 | AY945124 | GU245637 | 1 |
| Neolarrini | *Neolarra orbiculata* | N.A. | MK919620 | GU245029 | GU245205 | GU245333 | GU245486 | GU245661 | 1 |
|  | *Biastes truncatus* | D | MK919567 | GU244981 | GU245189 | HM211844 | GU245472 | GU245645 | 1 |
|  | *Neopasites cressoni* | D | MK919621 | GU245017 | GU245188 | GU245323 | GU245471 | GU245644 | 1 |
| Hexepeolini | *Hexepeolus rhodogyne* | A | MK919597 | GU245028 | GU245204 | GU245332 | GU245485 | GU245660 | 1 |
| Nomadini | *Nomada roberjeotiana* | A | OM722171 | OM906187 | OM906164 | OM906109 | OM906136 | OM850362 | 3 |
|  | *Nomada japonica* | A | OM722164 | OM906181 | OM906157 | OM906103 | OM906129 | OM850356 | 2 |
|  | *Nomada lathburiana* | A | OM722166 | OM906183 | OM906159 | OM906105 | OM906131 | OM850358 | 3 |
|  | *Nomada succincta* | A | EU678368 | X | X | KC798375 | OM906142 | OM850368 | 4 |
|  | *Nomada comparata* | A | OM722155 | OM906173 | OM906148 | OM906094 | OM906120 | OM850348 | 2 |
|  | *Nomada goodeniana* | A | OM722161 | OM906179 | OM906154 | OM906100 | OM906126 | OM850353 | 4 |
|  | *Nomada distinguenda* | D | OM722156 | OM906174 | X | OM906095 | OM906121 | - | 4 |
|  | *Nomada discedens* | D | - | - | OM906149 | X | OM906143 | X | 4 |
|  | *Nomada okubira* | D | OM722170 | OM906186 | OM906163 | OM912460 | OM906135 | OM850361 | 2 |
|  | *Nomada nipponica* | A | OM722168 | OM912459 | OM906161 | OM906107 | OM906133 | - | 2 |
|  | *Nomada armata* | A | OM722152 | OM906171 | OM906145 | - | OM906117 | - | 3 |
|  | *Nomada ginran* | A | OM722160 | OM906178 | OM906153 | OM906099 | OM906125 | OM850352 | 2 |
|  | *Nomada aswensis* | D | OM722153 | OM906172 | OM906146 | OM906092 | OM906118 | - | 5 |
|  | *Nomada kaguya* | D | OM722165 | OM906182 | OM906158 | OM906104 | OM906130 | OM850357 | 2 |
|  | *Nomada flavoguttata* | A | OM722158 | OM906176 | OM906151 | OM906097 | OM906123 | OM850350 | 3 |
|  | *Nomada maculata* | A | FJ582396 | GU245030 | GU245206 | AF344609 | GU245487 | GU245662 | 1 |
|  | *Nomada signata* | A | KJ839435 | KF512695 | GU245207 | KC798377 | KF512704 | KF512709 | 1 |
|  | *Nomada ferruginata* | A | KJ839795 | KF512693 | KF512698 | KC798369 | KF512702 | KF512707 | 1 |
|  | *Nomada panzeri* | A | KJ839787 | KF512696 | KF512700 | KC812731 | KF512705 | KF512710 | 1 |
|  | *Nomada flava* | A | KT074067 | KF512692 | KF512697 | KC798367 | KF512701 | KF512706 | 1 |
|  | *Nomada leucophthalma* | A | HQ948036 | KF512694 | KF512699 | KC798372 | KF512703 | KF512708 | 1 |
|  | *Nomada alboguttata* | A | OM722151 | OM906170 | OM906144 | OM906091 | OM906116 | OM850346 | 3 |
|  | *Nomada fulvicornis jezoensis* | N.A. | OM722159 | OM906177 | OM906152 | OM906098 | OM906124 | OM850351 | 2 |
|  | *Nomada fervens* | A | OM722157 | OM906175 | OM906150 | OM906096 | OM906122 | OM850349 | 6 |
|  | *Nomada marshamella* | A | OM722167 | OM906184 | OM906160 | OM906106 | OM906132 | OM850359 | 3 |
|  | *Nomada striata* | A | OM722173 | OM906190 | OM906167 | OM906112 | OM906139 | OM850365 | 3 |
|  | *Nomada ruficornis* | A | OM722172 | OM906188 | OM906165 | OM906110 | OM906137 | OM850363 | 3 |
|  | *Nomada obscura* | A | OM722169 | OM906185 | OM906162 | OM906108 | OM906134 | OM850360 | 3 |
|  | *Nomada icazti* | N.A. | OM722163 | OM912458 | OM906156 | OM906102 | OM906128 | OM850355 | 2 |
|  | *Nomada harimensis* | N.A. | OM722162 | OM906180 | OM906155 | OM906101 | OM906127 | OM850354 | 5 |
|  | *Nomada calloptera* | A | OM722154 | OM912457 | OM906147 | OM906093 | OM906119 | OM850347 | 2 |
|  | *Nomada shirakii* | A | x | OM906189 | OM906166 | OM906111 | OM906138 | OM850364 | 5 |

* -, unpublished; x, unavailable.

A: Andrenidae; B: Apidae; C: Colletidae; D: Halictidae; E: Melittidae.

1: downloaded from NCBI; 2: Seoul National University; 3: Finnish Museum of Natural History, (*N. alboguttata*: <http://id.luomus.fi/GJAA.549#1>; *N. armata*: <http://id.luomus.fi/GJAA.551#1>; *N. flavoguttata*: <http://id.luomus.fi/GJAA.504#1>; *N. goodeniana*: <http://id.luomus.fi/GJAA.377#1>; *N. lathburiana*: <http://id.luomus.fi/GJAA.393#1>; *N. marshamella*: <http://id.luomus.fi/GJAA.366#1>; *N. obscura*: <http://id.luomus.fi/GJAA.509#1>; *N. roberjeotiana*: <http://id.luomus.fi/GJAA.572#1>; *N. ruficornis*: <http://id.luomus.fi/GJAA.492#1>; *N. striata*: <http://id.luomus.fi/GJAA.588#1>); 4: Jan Smit, personal collection; 5: Keiichi Otsui, personal collection; 6: Korea National Arboretum
