# Supplementary material for: Harrison’s rule corroborated for the body size of cleptoparasitic cuckoo bees (Hymenoptera: Apidae: Nomadinae) and their hosts
Source: Sci Rep. 2022 Jun 29;12:10984. doi: 10.1038/s41598-022-14938-9 (PMC9243014; doi:10.1038/s41598-022-14938-9)
Supplement: Supplementary file 3 — Supplementary Information 3. [file 41598_2022_14938_MOESM3_ESM.docx]

**Table S2. Primers and PCR conditions.**

| **Gene** | **Direction** | **Primer** | **Sequences**  **(5’ – 3’)** | **Reference** |
| --- | --- | --- | --- | --- |
| COI^1^ | F | LCO1490 | GGT CAA CAA ATC ATA AAG ATA TTG G | Hebert et al., 2003 |
|  | R | HCO2198 | TAA ACT TCA GGG TGA CCA AAA AAT CA | Hebert et al., 2003 |
| EF-1α^2^ | F | EmphF2For | GCC TGG GTA TTG GAT AAG CTG AA | Sipes and Wolf, 2001 |
|  | R | EmphF2Rev | TGG ATT GTT YTT RGA GTC ACC AG | Sipes and Wolf, 2001 |
| Nak^3^ | F | NaKfor2 | GCS TTC TTC TCB ACS AAC GCC GTY GAR GG | Cardinal et al., 2010 |
|  | R | NaKrev2 | ACC TTG ATR CCG GCY GAW CGG CAC TTG GC | Cardinal et al., 2010 |
| Opsin^4^ | F | Opsin-For3 | TTC GAY AGA TAC AAC GTR ATC GTN AAR GG | Almeida and Danforth, 2009 |
|  | R | Opsin-Rev | ATA NGG NGT CCA NGC CAT GAA CCA | Almeida and Danforth, 2009 |
| Pol Ⅱ^5^ | F | polfor2a | AAY AAR CCV GTY ATG GGT ATT GTR CA | Danforth et al., 2006 |
|  | R | polrev2a | AGR TAN GAR TTC TCR ACG AAT CCT CT | Danforth et al., 2006 |
| Wingless^6^ | F | Wg-Collet-For | CAC GTG TCB TCB GRG ATG MGR SAG GA | Almeida and Danforth, 2009 |
|  | R | Lep-Wg2a-Rev | ACT ICG CAR CAC CAR TGG AAT GTR CA | Almeida and Danforth, 2009 |
| COI 1^7^ | F | COI1F | TAT ATT ATT TTT GCT TTA TG | This study |
|  | R | COI1R | AAT CAA AAW CTA ATA TTA TTY ATW C | This study |
| COI 2^7^ | F | COI2F | ATA GTD ATA CCW TTT ATA ATT G | This study |
|  | R | COI2R | GAT AAT ART AAT AAA AYW GC | This study |
| COI 3^7^ | F | COI3F | GTW GAT TTD GCW ATT TTT TC | This study |
|  | R | COI3R | TAA ATG TTG RTA TAA AAT WGG RTC | This study |
| EF-1α 1^8^ | F | EF1F | TAC GTT ACY ATC ATT GAT GC | This study |
|  | R | EF1R | ATT TCT TCG AAT CGG CTT TC | This study |
| EF-1α 2^8^ | F | EF2F | TGG TAT TTC GAA AAA TGG ACA AAC C | This study |
|  | R | EF2R | CAT TTT CCT TCA GTT TTG CC | This study |
| EF-1α 3^8^ | F | EF3F | AAA ATT GGT GGT ATC GGA ACG G | This study |
|  | R | EF3R | AAC CAA CGT TGT CRC CGG GAA CAG C | This study |
| Nak 1^9^ | F | Nak1F | AAG GAG ATA CAC CAT TTC ATT CAC | This study |
|  | R | Nak1R | GTC GGC TTC GAT GAT CTG ATT G | This study |
| Nak 2^9^ | F | Nak2F | CGG ATG GCS GCG AAG AAT TGT TTA G | This study |
|  | R | Nak2R | CTC TCG GGT GCG CCC TTC ATC | This study |
| Nak 3^9^ | F | Nak3F | AGA TCG CCA CGY TGT GCA ACC GTG C | This study |
|  | R | Nak3R | TCG GGT GCG CCC TTC ATC ACC | This study |
| Wng 1^10^ | F | Wng1F | ATG GTG AGC AAC ACG GAC CG | This study |
|  | R | Wng1R | TAC CGT GAG TGC CGA GAA TGC | This study |
| Wng 2^10^ | F | Wng2F | TCT ACC TGG AGC CCT CGC CAC | This study |
|  | R | Wng2R | TCG TCA CCT CCT GCG TCT TGT AG | This study |
| Opsin 1^11^ | F | Opsin1F | GTT TAG CTG GTA AGC CAT TG | This study |
|  | R | Opsin1R | AAG TAT ACC CAC AAG CTG TAC | This study |
| Opsin 2^11^ | F | Opsin2F | CAA CGA ACT CCT TTT AGA TAT GTA C | This study |
|  | R | Opsin2R | CAT GAG AGC AAC CTG GAA TKA TCG C | This study |
| Pol Ⅱ 1^12^ | F | Pol1F | GTA AGA AAA ATG ACG AAG AGG | This study |
|  | R | Pol1R | ACA AAG TAT ACC CAT GAC AAG | This study |
| Pol Ⅱ 2^12^ | F | Pol2F | ACA TGA TAA GAA CTC ACA GTA CG | This study |
|  | R | Pol2R | ATC GTT CAA AAT TCT GTT TAC TTG | This study |
| Pol Ⅱ 3^12^ | F | Pol3F | ATT GGT ATC GGR GAC ACC ATT GC | This study |
|  | R | Pol3R | AAT CGT CCT TGA TGA AAT GTG GCA G | This study |

^1^PCR conditions LCO1490 / HCO2198: one cycle of 1 min at 94 °C; five cycles of 1 min at 94 °C, 1.5 min at 45 °C and 1.5 min at 72 °C; 35 cycles of 1 min at 94 °C, 1.5 min at 50 °C and 1 min at 72 °C and a final cycle of 5 min at 72 °C.

^2^PCR conditions EmphF2For / EmphF2Rev: 94°C for 1 min, 52°C for 1 min, 72°C for 1.5 min (35 cycles).

^3^PCR conditions NaKfor2 / NaKrev2: 94°C for 1 min, 56–62°C for 1 min, 72°C for 1.5 min (35 cycles).

^4^PCR conditions Opsin-For3 / Opsin-Rev: 94°C for 1 min, 57°C for 1 min, 72°C for 1min (35 cycles).

^5^PCR conditions polfor2a / polrev2a: 94°C for 1 min, 55–57°C for 1 min, 72°C for 1min (35 cycles).

^6^PCR conditions Wg-Collet-For / Lep-Wg2a-Rev: 94°C for 1 min, 55°C for 1 min, 72°C for 1min (35 cycles).

^7^PCR conditions Partial primers (COI 1–3): 94°C for 1 min, 45°C for 1 min, 72°C for 1min (35 cycles).

^8^PCR conditions Partial primers (EF-1α 1–3): 94°C for 1 min, 55°C for 1 min, 72°C for 1min (35 cycles).

^9^PCR conditions Partial primers (Nak 1–3): 94°C for 1 min, 50°C for 1 min, 72°C for 1min (35 cycles).

^10^PCR conditions Partial primers (Wng 1–3): 94°C for 1 min, 55°C for 1 min, 72°C for 1min (35 cycles).

^11^PCR conditions Partial primers (Opsin 1–3): 94°C for 1 min, 55°C for 1 min, 72°C for 1min (35 cycles).

^12^PCR conditions Partial primers (Pol Ⅱ 1–3): 94°C for 1 min, 55°C for 1 min, 72°C for 1min (35 cycles).
